# Clinical application of two-port laparoscopic surgery in sigmoid colon and upper rectal cancer resection

**DOI:** 10.3389/fonc.2023.1248280

**Published:** 2023-11-06

**Authors:** Feng Jiang, Mengmeng Ji, Fangtong Jin, Junfeng Liu, Xiaoping Liu

**Affiliations:** ^1^ Department of General Surgery, The First Affiliated Hospital of Gannan Medical University, Ganzhou, China; ^2^ Ganzhou City Key Laboratory of Colorectal and Anal Diseases Research, Ganzhou, China; ^3^ Gannan Medical University, Ganzhou, China

**Keywords:** two-port laparoscopic surgery, conventional laparoscopic surgery, sigmoid colon cancer, upper rectal cancer, clinical application

## Abstract

**Background:**

In the field of minimally invasive surgery, the two-port laparoscopic surgery is on the rise. This study investigated the safety and efficacy of two-port laparoscopic surgery (TLS) for resecting sigmoid colon and upper rectal cancers compared with conventional laparoscopic surgery (CLS).

**Methods:**

The clinical data of patients undergoing laparoscopic sigmoid colon cancer and upper rectal cancer resection at the Department of General Surgery of the First Affiliated Hospital of Gannan Medical College between July 2019 and January 2022 were retrospectively collected. Grouped according to different laparoscopic surgery. Based on the inclusion and exclusion criteria,A total of 81 patients were enrolled, of the 25 patients from the TLS group,and of the 56 patients from the CLS group. We mainly compared whether there were statistical differences between the two groups in terms of operative time, intraoperative bleeding, incision length, time to first ambulation, time to first flatus, time to first defecation, postoperative complication rate, and other surgical outcomes.

**Results:**

There was no statistical difference between the two groups in terms of baseline clinical characteristics (*P* > 0.05). In terms of the surgical outcomes, there were statistical differences in the total incision length (TLS: 6.21 ± 0.67 cm, CLS: 8.64 ± 1.08 cm, *P* < 0.001)), time to first ambulation (TLS: 2.0 ± 0.7 d, CLS:3.1 ± 0.9 d, *P* < 0.001), time to first flatus (TLS: 2.5 ± 0.8 d, CLS: 3.0 ± 0.8 d, *P* = 0.028), time to first defecation (TLS: 3.8 ± 1.3 d, CLS: 5.1 ± 2.1 d, *P* = 0.010), and time for liquid diet (TLS: 4.3 ± 1.4 d, CLS: 5.3 ± 1.9 d, *P* = 0.021). There was no statistical difference between the two groups in terms of the pathology (*P* > 0.05).

**Conclusion:**

In terms of safety, TLS in sigmoid colon and upper rectal cancer resection is comparable to CLS. However, its incision is smaller and more aesthetic, and it causes lesser trauma than CLS. Additionally, it is also superior to CLS in postoperative recovery.

## 1 Introduction

Colorectal cancer associated with genetic mutations has become one of the most common malignancies (1). Its primary treatment is to remove the malignant tumor surgically. In 1991, Jacobs M et al. (2) tried applying the laparoscopic technique for resecting colon cancer. After 30 years of development, laparoscopic colorectal cancer resection has now become the preferred surgical procedure. Furthermore, modified laparoscopic surgical approaches, such as single-incision laparoscopic surgery (3) (SILS), natural orifice endoscopic surgery (4) (NOTES), etc. are also flourishing. Temporarily, SILS cannot be promoted on a large scale in clinical practice because of its difficulty, long learning curve, interference among surgical instruments, and linear view of the laparoscope (5). Therefore, the two-port laparoscopic surgery (TLS), which added an operating trocar to the SILS, was introduced (6). The TLS reconstructs the surgical triangle. The interference between the surgical instruments and the difficulty of surgery is lesser than that of SILS. Additionally, the learning curve of TLS is shorter, and it has the advantage of being minimally invasive. However, the safety and efficacy of TLS still lack a large body of medical evidence (7). This study aimed to investigate the safety and efficacy of TLS in sigmoid colon and upper rectal cancer resection.

## 2 Clinical data and methods

### 2.1 Study object

In this study, the clinical data of 81 patients admitted to the Department of General Surgery of the First Affiliated Hospital of Gannan Medical College between July 2019 and January 2022 for resection of sigmoid colon cancer and upper rectal cancer were retrospectively collected and grouped according to different laparoscopic surgical methods: 25 cases were operated by the two-hole method laparoscopy; 56 cases were operated by the conventional method laparoscopy. The differences in baseline data between the two groups of patients were not statistically significant (**Table 1**).

**Table 1:** Baseline clinical characteristics.

	TLS (n = 25)	CLS (n = 56)	*P*
Age (years)	62.0 ± 10.1	60.8 ± 12.7	0.685
Gender,n(%)			0.712
Male	15 (60.0)	36 (64.3)	
Female	10 (40.0)	20 (35.7)	
BMI (kg/m^2^)	23.1 ± 3.0	22.2 ± 2.5	0.176
ASA grade,n(%)			0.952
I	3 (12.0)	6 (10.7)	
II	17 (68.0)	40 (71.4)	
III	5 (20.0)	10 (17.9)	
Preoperative comorbidities			0.884
Intestinal obstruction	1	8	
Gastrointestinal bleeding	1	0	
Hypoproteinemia	2	2	
Anemia	1	2	
Tumor location from the anal verge (cm)	19.3 ± 6.8	19.4 ± 8.0	0.971
Tumor Location,n(%)			0.517
Sigmoid colon	11 (44.0)	29 (51.8)	
Superior rectum^a^	14 (56.0)	27 (48.2)	

Continuous variables are described as the mean ± standard deviation (range); categorical variables are described as n (%).

CLS, conventional laparoscopic surgery; TLS, two-port laparoscopic surgery; BMI, Body Mass Index.

ASA, American Society of Anesthesiologists.

^a^Recto-sigmoid junction cancer is included in the statistics of upper rectal cancer.

All cases were performed by the same surgical team, which had experience in more than 100 laparoscopic resections for sigmoid colon and upper rectal cancers. All patients were informed of the possible surgical risks and signed an informed consent form for the surgery before the operation. The study was approved by the Medical Ethics Committee of the First Affiliated Hospital of Gannan Medical College (approval number: LLSC-2020120201).

### 2.2 Inclusion and exclusion criteria

The inclusion criteria for this study were as follows: (1) age < 90 years; (2) BMI <30 kg/M^2^; (3) tumor lesions biopsied by endoscopy and pathologically diagnosed as malignant; (4) preoperative colonoscopy suggesting that the tumor located in the sigmoid colon and upper rectum (colonoscopy suggesting that the distance from the lower edge of the tumor to the anal verge was 8-40 cm; (5) tumor diameter < 8 cm; (6) imaging assessment, preoperative T-stage of T1 and T4a; and (7) American Society of Anesthesiologists (ASA) grade I-III.

The exclusion criteria for this study were as follows: (1) patients with severe liver and kidney dysfunction; (2) patients with combined malignant tumors of other sites or multiple primary colorectal cancer; (3) patients with signs of intestinal perforation and peritonitis requiring emergency surgery; (4) patients with intraoperative tumor infiltration of the surrounding organs requiring joint resection of infiltrating organs or palliative resection; and (5) patients with conversion to open surgery; (6) patients who underwent a prophylactic ileostomy; and (7) on abdominal implantation and distant metastasis, such as liver and lung.

### 2.3 Surgical method

Both groups followed the principle of complete mesocolic excision (CME). Furthermore, All patients were placed under general anaesthesia with tracheal intubation and in a modified lithotomy position (the patient’s right leg was about 20° lower than the left). The laparoscopist and the lead surgeon were posited on the patient’s right side. The laparoscopist was posited on the patient’s head side, the instrumentation nurse on the patient’s left side, and the laparoscopic monitor between the patient’s legs.

#### 2.3.1 Preoperative preparation

There was no significant difference in the preoperative preparation between the two groups. The patients were given oral laxatives at night, cleaning enemas, and other bowel preparations. In addition, all patients were given a liquid diet one day before the surgery. Also, prophylactic antibiotics were given 30 min before the surgery.

#### 2.3.2 Two-port laparoscopic surgery approach

**Figure 1:**
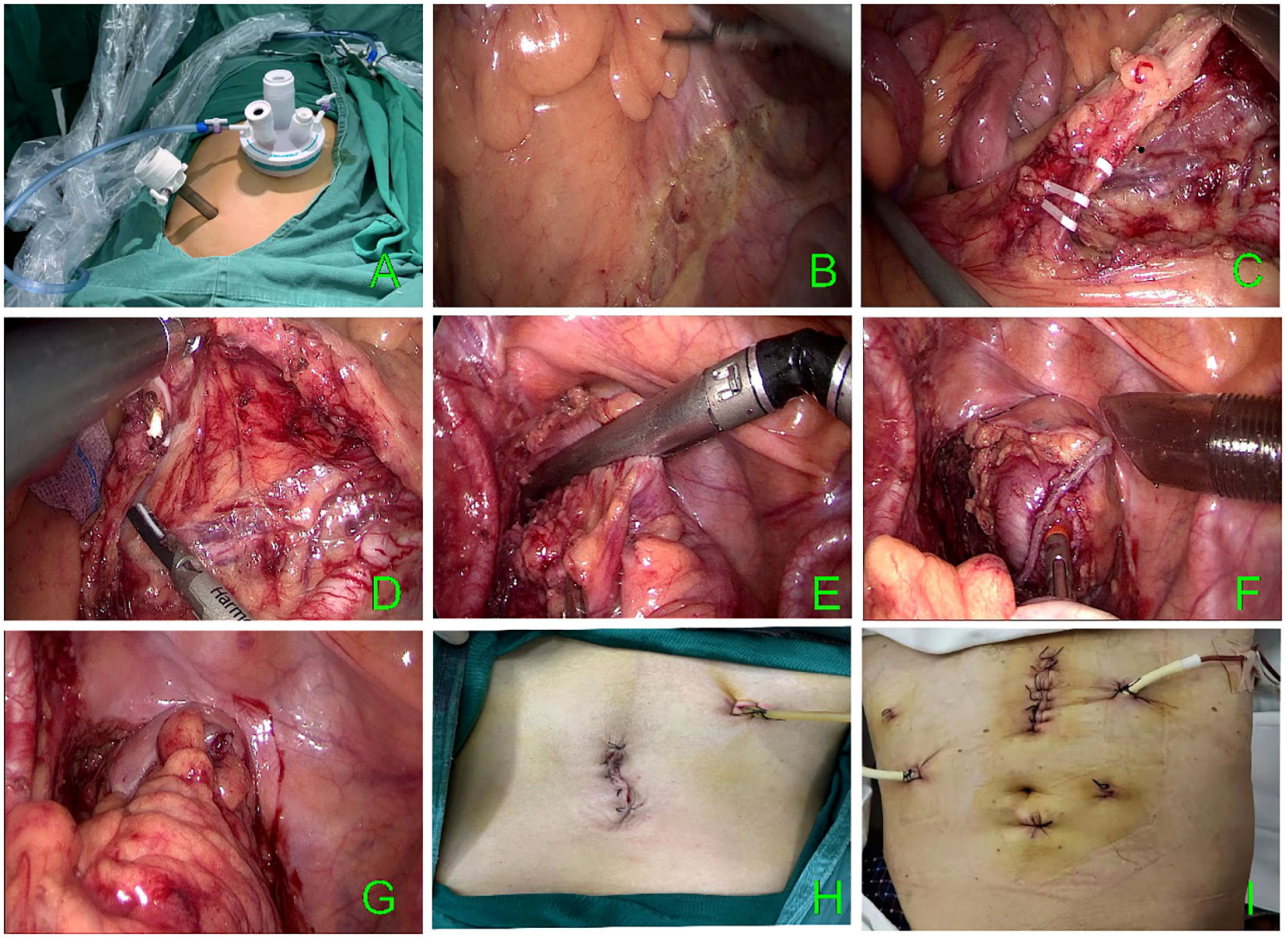
**(A)** Multiport device. **(B–G)** TLS surgical procedure. **(H)** TLS postoperative incision. **(I)** CLS postoperative incision.

A incision was made around the umbilicus entering the abdominal cavity. This was followed by placing a single-incision multiport device (Model: Surgaid 3D-70×150; contains 3 channels: laparoscopic body channel and lumpectomy instrument channel on both sides). with pneumoperitoneum pressure maintained at 15 mmHg. Subsequently, a 12 mm trocar was placed in the right lower abdomen at the McBurney point as the main operating trocar. (**Figure 1A**). The laparoscopic lens and intestinal forceps held by the lead surgeon’s left hand were inserted from the abdominal tract of the single-port platform and the instrument channel on the left side, respectively. The ultrasonic knife held by the right hand was inserted from the 12 mm trocar at the McBurney point. The abdominal cavity was routinely explored. The head-low, foot-high, right-tilted position was changed, and the small bowel was placed in the right iliac fossa and the superior colonic region to fully expose the operative area. Two pieces of small gauze for laparoscopy were placed in the right iliac fossa and the mesenteric root to increase the friction between the small bowel and the intestinal canal. This method also prevented the small bowel from falling into the operative area and causing interference. Using the middle approach, the projection area of the inferior mesenteric vein was identified. The lead surgeon’s left hand pulled the sigmoid mesentery cephalad and laterally. After tensioning the sigmoid mesentery, the surgeon entered at the level of the sacral capsule. Then, the left Toldt gap was identified, and it was entered for an extension. The ultrasonic knife was fully utilized to sharply and bluntly extend the left Toldt space up to the root of the inferior mesenteric artery (IMA), left to the lateral genital vessels, and down to the posterior rectal space. A root dissection of the IMA or preservation of the left colonic artery (LCA) was considered, depending on the patient’s condition, intestinal blood supply, and other factors. After fully exposing the IMA, if it was severed at the root, the pedicle of the IMA and the abdominal aorta were pulled to an angle of 45° with the left hand of the main knife, and the IMA was double ligated at a distance of 1.5 cm from the root. If the LCA was preserved, the IMA should be dissected below the LCA bifurcation to preserve the blood supply to the colon. The submesenteric artery was dissected below the LCA bifurcation to preserve the colonic blood supply. Then, the mesentery was dissociated to the left to expose the inferior mesenteric vein (IMV) which was then ligated. After dissecting the vessels, the left lateral peritoneum which was met on the right side was dissected laterally. The mesentery was cut up to 10 cm above the tumor. Proper care was taken to protect the vessels at the edge of the intestinal tube; the intestinal tube was naked at 5 cm from the lower edge of the tumor and cut with an electric linear cutting closure (60mm Endo-gia Blue). The pneumoperitoneum was closed, the single-incision multiport device was removed, the intestinal tube was removed at the incision (if the transverse diameter of the tumor was too large to be removed for preservation, the incision needed to be enlarged appropriately), the specimen was resected, and the ruffle suture was placed into a 29-gauge tubular anastomosis anvil (Ethicon CDH 29A). The pneumoperitoneum was re-established. Then, the tubular anastomosis was placed through the anus under direct laparoscopic view. Subsequently, an end-to-end intestinal anastomosis was performed without tension on both ends of the intestine. The anastomosis was strengthened, the abdominal cavity was washed with sterilized water, and the pelvic drainage tube was placed through the incision given at the McBurney point in the lower right abdomen. The abdominal cavity was closed. The procedure of the operation is shown in **Figures 1B–G**, and the abdominal incision after the operation is shown in **Figure 1H**.

#### 2.3.3 Conventional laparoscopic surgical approach

The patient was given a modified lithotomy position with routine tracheal intubation, general anesthesia, and sterile towel laying. A 10 mm incision was given at the umbilicus for observation; 12 mm and 5 mm trocar incisions were given in the left abdomen; and two 5 mm trocar incisions were given in the right abdomen. The positions of the main surgeon, scope hand, and instrument nurse were the same as those in the single-port plus group. The assistant was positioned on the patient’s right side. After the dissection of the intestinal canal, the specimen was taken out through an incision in the lower abdomen in the conventional laparoscopic group; then the specimen was anastomosed laparoscopically. Finally, drainage tubes were placed on each side. The postoperative abdominal incision is shown in **Figure 1I**.

## 3 Observed indicators

(1) Surgical outcomes: total operation time, blood loss, auxiliary incision length, the total length of incision, time to first ambulation, time to first ambulation, time to first flatus, time to first defecation, time for a liquid diet, days of drainage tube removal, postoperative complications (anastomotic leakages, anastomotic bleeding, incision infection, lymphatic leak, intestinal obstruction, lung infection, etc.), days of hospitalization after surgery (2) Pathologic outcomes: pathologic TNM stage, depth of tumor invasion (T-stage), distal resection margin (DRM), proximal resection margin (PRM), harvested number of lymph nodes, etc.

## 4 Statistical processing methods

The SPSS 21.0 software was used. The count data were described by frequencies or percentages. They were compared and analyzed by using the chi-square test. Fisher’s exact probability method was used for theoretical frequencies < 1. The measurement data were described by the sample mean ± standard deviation ( $\bar{x}$ ± s) and compared and analyzed by using the paired-sample t-test. *P* < 0.05 was considered a statistically significant difference.

## 5 Results

The differences in the baseline clinical characteristics between the two groups were not statistically significant (*P* > 0.05) (**Table 1**). In terms of total operative time and blood loss in both groups, there were no statistically significant differences in the data between the two groups (*P* > 0.05). In terms of auxiliary incision length, no statistically significant difference was noted between the TLS (4.88 ± 0.64 cm) and the CLS groups (5.14 ± 1.01 cm), *P* = 0.243. However, for the total incision length (TLS: 6.21 ± 0.67 cm CLS: 8.64 ± 1.08 cm), time to first ambulation (TLS: 2.0 ± 0.7 d CLS: 3.1 ± 0.9 d), time to first flatus (TLS: 2.5 ± 0.8 d CLS: 3.0 ± 0.8 d), time to first defecation (TLS: 3.8 ± 1.3 d CLS: 5.1 ± 2.1 d), and the time for liquid diet (TLS: 4.3 ± 1.4 d CLS: 5.3 ± 1.9 d); the differences were statistically significant (*P <* 0.05). For days of drainage tube removal, postoperative complications, and postoperative hospital days, the differences were not statistically significant (*P* > 0.05). There were no statistically significant differences between the two groups in terms of tumor diameter, pathological TNM stage, depth of tumor infiltration (T-stage), proximal resection margin, distal resection margin, and harvested number of lymph nodes (*P* > 0.05); The surgical and pathological outcomes are presented in **Tables 2**, **3**.

**Table 2:** Surgical outcomes.

	TLS (n = 25)	CLS (n = 56)	*P*
Total operation time^a^ (min)	173.8 ± 46.0	177.3 ± 59.0	0.793
Blood loss (ml)	47.4 ± 20.7	57.9 ± 28.9	0.105
Auxiliary incision length^b^ (cm)	4.88 ± 0.64	5.14 ± 1.01	0.243
The total length of incisions^c^ (cm)	6.21 ± 0.67	8.64 ± 1.08	< 0.001
Time to first ambulation (days)	2.0 ± 0.7	3.1 ± 0.9	< 0.001
Time to first flatus (days)	2.5 ± 0.8	3.0 ± 0.8	0.028
Time to first defecation (days)	3.8 ± 1.3	5.1 ± 2.1	0.010
Time for liquid diet (days)	4.3 ± 1.4	5.3 ± 1.9	0.021
Days of drainage tube removal^d^ (days)	10.2 ± 3.8	9.4 ± 2.7	0.332
Postoperative complications (n)			0.771
Anastomotic leakages	0	1	
Anastomotic bleeding	0	1	
Incision infection	1	1	
Lymphatic leak	0	1	
Intestinal obstruction	1	0	
Lung infection	1	0	
Days of hospitalization after surgery(days)	13.8 ± 4.0	12.8 ± 3.9	0.342

Values are presented as sample mean ± standard deviation ( $\bar{x}$ ± s).

^a^Total operation time refers to the time from the start of skin removal to the closure of the abdominal incision.

^b^Auxiliary incision refers to the specimen removal incision for both sets of procedures, which is also a single-hole platform placement incision in TLS.

^c^Total length of the incision refers to the length of the specimen removal incision plus the length of the incision for all trocar punctures in the abdomen.

^d^The day of drainage tube removal in the TLS group is the day of drainage tube removal placed in the right lower abdomen, while in the CLS group, one drainage tube is placed bilaterally in a few patients, and the time of drainage tube removal in the right lower abdomen is also counted here.

**Table 3:** Pathologic outcomes.

	TLS (n = 25)	CLS (n = 56)	*P*
tumor diameter (cm)	4.12 ± 1.30	4.34 ± 1.75	0.565
Pathologic TNM stage,n(%)			0.212
I	2 (8.0)	9 (16.1)	
II	14 (56.0)	20 (35.7)	
III	9 (36.0)	27 (48.2)	
Depth of tumor invasion,n(%)			0.173
T1	1 (4.0)	4 (7.1)	
T2	2 (8.0)	6 (10.7)	
T3	16 (64.0)	21 (37.5)	
T4a	6 (24.0)	25 (44.6)	
PRM (cm)	5.13 ± 2.21	4.74 ± 2.13	0.458
DRM (cm)	3.50 ± 1.76	3.86 ± 2.26	0.474
Harvested no. of LN (n)	16.6 ± 5.5	18.2 ± 6.9	0.321

Continuous variables are described as the mean ± standard deviation (range); categorical variables are described as n (%).

TNM, tumor-node-metastasis; LN lymph node; PRM, proximal resection margin; and DRM, distal resection margin.

## 6 Discussion

Since its introduction in 2012, TLS has received a lot of attention from scholars. Also, several large clinical research centers have started relevant clinical studies and published some of their research results (8, 9). Some clinical research centers have applied TLS for gastric cancer (10), right hemicolectomy, left hemicolectomy, sigmoid colon cancer, rectal cancer, and other sites. Typically, only the inferior mesenteric artery and vein need to be ligated for lymph node dissection in patients with cancer of the sigmoid colon and upper rectum; moreover, no splenic flexion mobilization is required, and satisfactory distal resection margins can be obtained without excessive mobilization of the rectum outside the peritoneum reflection. As a result, the surgical procedure is easier to learn. For now, our center only performs TLS surgery in sigmoid colon and upper rectal cancers.

The clinical experience of some clinical research centers revealed that elderly and obesity are independent risk factor for postoperative complications of laparoscopic radical resection of right-sided hemicolon cancer after complete mesocolic excision (CME) (11). And tumor diameter were independent predictors of operative time in laparoscopic anterior resection for rectal cancer (12). Therefore, TLS surgery was performed in patients with BMI ≤ 30 kg/m^2^, tumor diameter < 5 cm, and preoperative evaluation of T4b or less (13). However, our study revealed that in two-port laparoscopic resection of the sigmoid colon and upper rectal cancer, excessive omental and mesenteric hypertrophy and deep tumor infiltration indeed caused excessive interference with the anatomical level recognition and the surgical plane development due to excessive BMI. This made the surgical progress slow or even required the usage of an additional trocar. The increase in tumor weight due to excessive tumor size also interfered with surgery considerably. However, this interference can be solved to a certain extent with the increase in the operator’s experience. At our center, a patient with preoperative enhanced CT suggesting a tumor size of about 8 cm, tumor infiltration depth of T4a, and BMI of 20.2 kg/m^2^ with a recto-sigmoid junction tumor was successfully treated with TLS without using an additional trocar adjuvant. However, the operating time for this patient increased correspondingly. At our center, the average total operation time for sigmoid colon and upper rectal cancer was 173.8 min, but the total operation time for this patient was 240 min. Since then, our center also operated on four patients with a tumor diameter of > 5 cm, whose BMI were 18.8 kg/m^2^, 20.9 kg/m^2^, 22.7 kg/m^2^, and 25.2 kg/m^2^, and the depth of tumor infiltration was below T4b. Therefore, our center considers that in patients with relatively small or moderate BMI, whose omental and mesenteric adipose tissues cause a decreased impact on surgery, and with experienced surgeons, the indication of tumor diameter can be moderately relaxed for selected patients. However, whether prolonged operative time due to the enlarged tumor has an impact on the patient’s postoperative recovery needs further exploration.

There is enormous evidence suggesting that laparoscopic colorectal cancer resection is feasible, with adequate surgical quality and faster postoperative recovery, more aesthetic incisions, etc. (14) The operative time is an important indicator of the difficulty of laparoscopic surgery (15). For example, Kang et al. (16) reported that the average operation time of 93 single-port laparoscopic and 88 conventional laparoscopic colorectal cancer surgeries were 189 min and 170 min, respectively. Watanabe (17) et al. reported that the average operation time for 100 colorectal cancer procedures was 156 min and 162 min for SILS and CLS, respectively. In these two clinical studies, there was no statistically significant difference between single-incision laparoscopic colorectal cancer surgery and conventional laparoscopic colorectal cancer surgery in terms of the average operation time (*P* > 0.05). Z J Wu et al. (18) retrospectively analyzed 30 patients who underwent two-port laparoscopic sigmoid colon and upper rectal cancer with an average operation time of 125 min. Wang Y et al (8) showed that the average intraperitoneal operation time was 66.2 ± 26.9 min for TLS and 76.3 ± 28.2 min for CLS. Although there was no statistically significant difference between the two groups, the intracavitary operation time was slightly shorter in the TLS than in the CLS. Studies have confirmed that the familiarity among laparoscopic team members will influence the procedure time (19), Poorly coordinated surgical assistants can even cause unnecessary disruptions to the operation.Thus, compared to CLS, the role of a surgical assistant in the TLS can be replaced by the experienced left hand of the surgeon in suitable conditions and even reduce the operation time.

Surgical safety, which was the focus of this study, was measured mainly by intraoperative and postoperative complications and postoperative tumour morbidity and mortality. With regard to intraoperative blood loss, some studies have indicated a significant increase in blood loss in the CLS group, mainly due to the loss of the inferior epigastric artery in one case resulting in increased blood loss (8). However, in our study, there was no significant difference in intraoperative blood loss between the two in TLS and CLS. In terms of postoperative complications, it has been suggested that the use of more trocars may increase the likelihood of trocar-related complications such as bleeding and herniation (20), but this did not occur in any patient in our study (trocar hernia). In our study, two patients in the CLS group had third-grade complications, one anastomotic leakages. and one anastomotic bleeding, and none of these occurred in the TLS group. However, there was no difference in the total number of complications between the two groups, similar to the findings of most studies (21–25). Therefore, the safety of TLS in the perioperative period was comparable to that of CLS. However, whether there is a difference in postoperative tumour morbidity and mortality needs to be confirmed by further follow-up data.

Although the extent of surgical resection and lymph node dissection was the same between the two groups, when we analysed the patients’ postoperative recovery, we found that in the TLS group, Time to first ambulation (days), Time to first flatus (days), Time to first defecation (days) Time for liquid diet (days) were all shorter than in the CLS group, and the differences were statistically significant. Postoperative recovery was better in the TLS group than in the CLS. Moreover, some studies have shown that CLS has a higher VAS (Visual analogue scale) score at POD3 (POD: postoperative day) than TLS (26); we speculate that this may be due to the fact that TLS used fewer trocars resulting in a faster postoperative recovery, but the exact reason for this remains to be further verified.

For the cosmetic results, we measured the total length of the trocar needle and incision. The difference in auxiliary incision length was not statistically different, but the total incision length was less in the TLS group than in the CLS group.

Our study has several limitations.firstly, this study is a single-centre study with a limited number of cases, which still needs to be validated with a larger sample size and multi-centre data; secondly, this study is a retrospective study with potential confounding factors, which needs to be further explored in prospective studies; lastly, this study focuses on collecting and analysing preoperative and intraoperative relevant indexes, and there is a lack of postoperative follow up of long-term tumour outcomes in both groups,which is also an area that our team needs to improve and study later.

## 7 Conclusions

In summary, in sigmoid colon and upper rectal cancer resection, the TLS retained the aesthetic incision and other advantages of SILS, reconstructed the surgical triangle, solved the problem of conflicting main operating instruments, and reduced its surgical difficulty. In addition, it also resulted in smaller and more aesthetic incisions, lesser trauma, and faster postoperative recovery than CLS. This is in consistent with the conclusions of other scholars (14). It was superior to CLS in terms of short-term postoperative efficacy. And our center considers that in patients with relatively small or moderate BMI, and with experienced surgeons, the indication of tumor diameter can be moderately relaxed for selected patients in TLS.

## Author contributions

(I) Conception and design: XL, FJia; (II) Administrative support: XL; (III) Provision of study materials or patients: XL; (IV) Collection and assembly of data: MJ, FJin; (V) Data analysis and interpretation: MJ, FJia, JL; (VI) Manuscript writing: All authors; (VII) Final approval of manuscript: All authors.
